# The clinical impact of bacterial co-infection among moderate, severe and critically ill COVID-19 patients in the second referral hospital in Surabaya

**DOI:** 10.12688/f1000research.31645.2

**Published:** 2021-03-29

**Authors:** Tri Pudy Asmarawati, Alfian Nur Rosyid, Satriyo Dwi Suryantoro, Bagus Aulia Mahdi, Choirina Windradi, Prastuti Asta Wulaningrum, Muhammad Vitanata Arifijanto, Bramantono Bramantono, Erwin Astha Triyono, Musofa Rusli, Brian Eka Rachman, Erika Marfiani, Pepy Dwi Endraswari, Usman Hadi, Kuntaman Kuntaman, Nasronudin Nasronudin

**Affiliations:** 1 Department of Internal Medicine, Faculty of Medicine, Universitas Airlangga, Surbaya, East Java, 60115, Indonesia; 2 Universitas Airlangga Hospital, Surabaya, East Java, 60115, Indonesia; 3 Dr. Soetomo General Teaching Hospital, Surabaya, East Java, 60286, Indonesia; 4 Department of Pulmonology and Respiratory Medicine, Faculty of Medicine, Universitas Airlangga, Surabaya, East Java, 60115, Indonesia; 5 Department of Clinical Microbiology, Faculty of Medicine, Universitas Airlangga, Surabaya, East Java, 60286, Indonesia

**Keywords:** Bacterial infection, COVID-19, SARS-CoV-2, antibiotics

## Abstract

**Background:** Data on the prevalence of bacterial co-infections among COVID-19 patients are limited, especially in our country, Indonesia. We aimed to assess the rate of bacterial co-infections in hospitalized COVID-19 patients and report the most common microorganisms involved and the antibiotic use in these patients.

**Methods:** This study is a retrospective cohort study,among COVID-19 adult patients admitted to Universitas Airlangga Hospital Surabaya from 14 March-30 September 2020. The bacterial infection is defined based on clinical assessment, laboratory parameters, and microbiology results.

**Results:** A total of 218 patients with moderate to critical illness and confirmed COVID-19 were included in this study. Bacterial infection was confirmed in 43 patients (19.7%). COVID-19 patients with bacterial infections had longer hospital length of stay (17.6 6.62 vs 13.317.12), a higher proportion of respiratory failure, intensive care treatment, and ventilator use. COVID-19 patients with bacterial infection had a worse prognosis than those without bacterial infection (p<0.04). The empirical antibiotic was given to 75.2% of the patients. Gram-negative bacteria were commonly found as causative agents in this study (n = 39; 70.37%).

**Conclusion:** COVID-19 patients with bacterial infection have a longer length of stay and worse outcomes. Healthcare-associated infections during intensive care treatment for COVID-19 patients must be carefully prevented.

## Introduction

Coronavirus disease (COVID)-19 has experienced an increase in 2,995,758 positive cases and 204,987 deaths in distribution areas of more than 213 countries ^1^. In Indonesia, until November 2020, there were 522,581 confirmed cases of COVID-19 with 68,604 active cases, 437,456 recovered cases, and 16,521 (3%) deaths. According to national data, among the total number of cases, East Java is the second-highest prevalence of 60,190 (11.6%). As of November 26, 2020, the cumulative data on confirmed COVID-19 patients in Surabaya were 16,763 with 1204 (7.03%) deaths ^1^.

Data regarding secondary respiratory infection in COVID-19 patients are still limited in Indonesia even though the cases distribution is still worldwide. Several reports suggest that secondary infection will impact the patients survival and increase intensive care unit (ICU) treatment ^2^. COVID-19 pneumonia itself is also related to increasing ICU care, secondary infection rate, and higher invasive treatment ^3^. Co-infection of SARS-CoV-2 and other microorganisms such as viruses, bacteria, and fungi is an essential factor in COVID-19 treatment since this condition may raise the difficulty in diagnosis, treatment, and the prognosis, also increasing the mortality ^4, 5^. Upper respiratory tract infection, a typical manifestation of COVID-19, is challenging to differentiate from other causes of pneumonia.

There are various types of co-infection in COVID-19; such as: 1) secondary SARS-CoV-2 following bacterial infection or colonization; 2) mixed infection between viral and bacterial pneumonia infection; 3) secondary bacterial superinfection following SARS-CoV-2 infection. The mechanisms underlying those types of infection are very dependent on the onset and involving complex interactions between three different agents (virus, host, and bacteria). Immune response towards SARS-CoV-2 infection only differs with mixed infection to bacteria or viral pneumonia. Therefore, it could be hypothesized that any co-infection will worsen the outcome and severity of COVID-19 ^6^.

Although several studies have investigated the epidemiological and clinical characteristic of COVID-19, information regarding SARS-CoV-2 infection with secondary infection are limited ^7^. This study aims to describe bacterial co-infection and antibiotic use among patients who confirmed SARS-CoV-2 infection from moderate to critically ill manifestation in Universitas Airlangga hospital Surabaya.

## Methods

### Study design and population

This study is a retrospective cohort study, total sampling, among COVID-19 adult patients admitted to Universitas Airlangga Hospital Surabaya. This hospital is an academic hospital and also a referral hospital for COVID-19 management in Surabaya, East Java Region. We included cases of moderate to critically ill COVID-19 patients between 14 March and 30 September 2020 that were admitted in the intensive care unit or high-care unit. COVID-19 diagnosis was made based on World Health Organization (WHO) guidelines ^8^ and the Indonesian Ministry of Health guidelines ^9^. Confirmed COVID-19 patients were proven by oropharyngeal and nasopharyngeal swabs SARS-CoV-2 PCR (polymerase chain reaction). Bacterial infection was defined based on clinical assessment, laboratory parameters, and inflammatory parameters (C-reactive protein (CRP) and procalcitonin). Bacterial co-infection of SARS-CoV-2 defines if the culture samples were taken at patient presentation to the hospital or < 48 hours admission, while secondary bacterial infection defined if the culture samples were taken > 48 hours of admission. The bacterial causative agents were extracted from data of microbiology, that were identified by Microbiology automated machine Vitek-2 compact, as a routine procedure in this hospital.

This study was approved by the Ethical Committee of Universitas Airlangga Hospital (171/KEP/2020). Written informed consent was obtained from all participants prior to the start of the study.

### Data collection

Data were taken from medical records and microbiology reports from the laboratory. Incomplete medical records were excluded. Clinical characteristics were divided according to the severity of the disease. Moderate case definitions are: 1) clinically sign of pneumonia (fever, cough, dyspnea, tachypnea); 2) Oxygen saturation 93% free air. Severe case definitions are if there were clinically sign of pneumonia, and one of the following: 1) respiration rate >30 times per minute, or 2) severe respiratory distress, or 3) oxygen saturation < 93% free air. Critically ill cases defined if there were acute respiratory distress syndrome (ARDS), sepsis, and septic shock ^10^. Culture examination was performed when there was suspicion of bacterial infection or sepsis. The sample for culture was taken from blood, urine, and respiratory tract.

### Statistical analysis

Data were analyzed with SPSS version 24.0 (Chicago, IL, USA) ^11^. Descriptive statistics included categorical variables reported as number (percentage) and continuous variables as mean (standard deviation). For missing data, we used listwise deletion or univariable and multivariable analysis. Chi-square test and Mann-Whitney test were used accordingly to the type of variable. Categorical variables were shown as number (%) and continuous variables as mean (standard deviation) or median (range) depending on whether the data are normally distributed or not. Statistical significance was assessed by means of chi-squared for dichotomous variables, or by means of the two independent sample t-test or the Mann-Whitney U test for continuous variable depending on whether the data are normally distributed or not.

## Results

### Patient characteristics

From March 14 until September 30, 2020, a total of 218 patients confirmed for SARS-CoV-2 infection were admitted to Universitas Airlangga Hospital Surabaya from moderate to critically ill condition. Patients characteristic symptoms were defined according to their severity. Clinical characteristics and main comorbidities are detailed in Table 1. The median age of the study subject was 52.45 (14.44) years, and 55.05% of patients were male. According to disease severity, the number of patients with moderate, severe, and critically ill manifestations were 126 (57.8%), 40 (18.3%), and 52 (23.9%), respectively. Diabetes and hypertension were the most common comorbidity, in 34.4% and 29.9% of patients, respectively. Among all subjects, patients that were critically ill 7.3% manifested respiratory failure (p <0.05), 23% were on a ventilator (p <0.05) and in 7.8% were in sepsis (p = 0.006). Critically ill patients had the longest length of stay (mean 16.89 9.4 days). Bacterial infection was confirmed in 43 patients (19.7%); 16 patients were in critically ill condition. Among COVID-19 patients with a bacterial infection, we divided them into two categories, bacterial co-infection (23%) and secondary bacterial infection (77%).

**Table 1.** Baseline Characteristics of Patients.

	Severity COVID-19 (n=218)						Total (n.%)		p Value
	Moderate		Severe		Critical Ill				
	126		40		52		218	100	
**Sex**	57.80		18.35		23.85			100	
Male (n=120)	69		27		24		120.00	55.05	0.12
Female (n=98)	57		13		28		98.00	44.95	
**Age(Mean/SD)**	51.04	15.32	53.50	14.63	55.02	11.65	52.45	14.44	0.21
**Comorbidities**									
DM (n.%)	41	18.8	11	5	23	10.6	75	34.4	0.2
HT (n.%)	35	16.1	6	2.8	24	11	65	29.9	0.004
Geriatric (> 60 years) (n.%)	31	14.2	12	5.5	16	7.3	59	27	0.63
Heart disease (n.%)	7	3.2	4	1.8	3	1.4	14	6.4	0.83
Stroke (n.%)	7	3.2	1	0.5	3	1.4	11	5.1	0.72
CKD (n.%)	5	2.3	2	0.9	2	0.9	9	4.1	0.95
Smoker (n.%)	5	2.3	2	0.9	0	0	7	3.2	0.1
COPD (%)	1	0.4	0	0	0	0	1	0.4	0.1
Liver disease (n.%)	0	0	0	0	1	0.5	1	0.5	0.2
Respiratory Failure (n.%)	0	0	0	0	43	19.7	43	19.7	<0.05
Ventilator (n.%)	0	0	0	0	23	10.5	23	10.5	<0.05
Sepsis (n.%)	0	0	0	0	17	7.8	17	7.8	0.006
**Type of bacterial infection**									
< 48 hours (n.%) (Co-infection)	4	1.8	3	1.4	6	2.8	13.0	6.0	0.52
> 48 hours(n.%) (Secondary Infection)	22	10.1	16	7.3	16	7.3	54.0	24.8	
**Bacterial Infection (n.%)**	15	6.9	12	5.5	16	7.3	43.0	19.7	0.55
**Antibiotic use (n.%)**	90	41.3	44	20.2	30	13.8	164.0	75.3	
**Symptom and Duration**									
Dypsnea (%.days)	19.27	4.00	7.34	6.00	14.68	3.20		41.29	
Fever (%. days)	16.97	6.40	3.21	6.00	2.75	8.80		22.93	
Cough (%.days)	7.34	4.60	3.21	10.60	0.92	3.00		11.47	
Malaise (%. days)	7.34	2.60	0.92	3.50	2.29	6.80		10.55	
Nausea and Vomiting (%. days)	1.38	6.30	0.92	4.00	0.46	7.00		2.76	
Diarrhea (%. days)	0.92	3.50	0	-	0	-		0.92	
Headache (%. days)	0.92	2.00	0	-	0.92	3.00		1.84	
Anosmia (%. days)	0.46	2.00	0	-	0	-		0.46	
Joint pain (%. days)	0.46	4.00	0.46	7.00	0	-		0.92	
Chest pain (%. days)	0.46	2.00	0.92	1.00	0	-		1.38	
Loss of consciousness (%. days)	0	0.00	0.92	1.50	1.84	2.30		2.76	
**Vital sign**									
Systolic Pressure (mean. SD)	133.5	20.62	125.7	20.1	131.63	27.42			0.161
Diastolic Pressure (mean.SD)	87.9	60.47	78.2	8.6	79.96	13.92			0.104
Respiration Rate (mean. SD)	23.34	3.76	24.73	3.52	28.92	6.4			< 0.05
Temperature (mean.SD)	37.38	4.87	36.88	0.91	36.73	0.75			0.512
Oxygen saturation (mean. SD)	94.2	3.97	88.05	9.5	88.24	11.29			< 0.05
**Laboratory**									
Leucocyte (10 ^3^/uL; mean. SD)	8.52	4.26	10.01	5.78	11.01	6.98			0.014
Neutrophil (%. mean. SD)	69.86	18.98	71.26	23.43	81.16	6.71			0.001
Lymphocyte (%. mean. SD)	16.99	10.32	12.76	8.83	11.86	5.24			0.001
Neutrophil-Lymphocyte Ratio (NLR) (mean. SD)	8.65	30.56	8.15	6.02	8.94	5.44			0.98
Thrombocyte (10 ^3^/uL; mean. SD)	260.13	114.39	288.97	137.74	289.46	120.21			0.213
C-Reactive Protein (mg/L; mean. SD)	11.15	29.27	16.6	35.19	23.37	50.1			0.122
Procalcitonin (ng/ml; mean. SD)	0.055	0.18	1.18	6.58	1.62	5.55			0.033
Basal Urea Nitrogen (mg/dl; mean.SD)	16.75	23.2	16.16	11.44	29.35	34.88			0.007
Creatinine (mg/dl; mean. SD)	1.3	2.14	1.72	3.96	1.9	3.3			0.4
Aspartate aminotransferase (u/L; mean. SD)	25.11	28.1	31.78	27	90.9	64.58			0.78
Alanine aminotransferase (u/L; mean. SD)	28.1	43.65	27	34.1	64.58	135.6			0.01
PaO2:FiO2 ratio (n=71)(mean. SD)	242.53	70.62	160.67	90.54	132.78	57.031			0.005
**Length of stay (mean/SD)**	12.97	6.7	15.61	7.1	16.89	9.4	14.12	7.21	0.03
**Outcome**									
Discharge (n.%)	115	52.7	38	16.4	30	13.8	183.0	82.9	<0.05
Death (n.%)	0	0	2	0.9	19	8.7	21.0	9.6	<0.05

Characteristic symptoms and laboratory result are shown in Table 1. The most common symptoms in all severity were dyspnea, fever, cough, and malaise. In patients who were moderate, severe, and critically ill, symptoms cough (7.34%, 3.21%, 0.92%), fever (16.97%, 3.21%, 2.75%) and dyspnea (19.27%, 7.34%, 14.68%) respectively. Other symptoms recorded were nausea, vomiting, diarrhea, headache, joint pain, chest pain, and loss of consciousness, but only affected the minority of the patients. From vital sign examination, two variables had a significant difference for moderate, severe, and critically ill patients; respiratory rate (23.34, 24.73, 28.92; p <0.05) and oxygen saturation (SaO _2_) (94.2, 88.05, 88.24; p <0.05) respectively. Some symptoms vary in duration between groups. Severe patients complained of cough symptoms for an average of 10.6 days, longer than those in the moderate or critically ill group. The duration of fever was almost similar between groups, namely 68.8 days.

From the laboratory findings, the mean leucocyte count (p = 0.014), neutrophil count (p = 0.001), procalcitonin (p = 0.033), basal urea nitrogen (BUN) (p = 0.007), alanine aminotransferase (ALT) levels (p = 0.01) were significantly higher in either severe or critically ill COVID-19 patients than moderate cases. We also found a lower lymphocyte count (p = 0.001) and PaO _2_: FiO _2 _ratio (p = 0.005) in either severe or critically ill COVID-19 patients than moderate cases. The procalcitonin level significantly increased in severe and critical illness conditions. The CRP level also increased but was not statistically significant. The majority of patients (82.9%) were recovered and discharged from the hospital, while 9.6% of the patient died. Most of the patients who died were in critically ill condition at presentation to hospital. 

### Bacterial infections

Bacterial infection was confirmed in 43 patients (19.7%) (see Table 2). There were no sex differences between bacterial infection and no bacterial infection patients. Patients with a bacterial infection have an older mean age than no bacterial infection, although, among elderly patients, there were no differences in bacterial infection rate. We documented one patient with chronic obstructive pulmonary disease (COPD) and one with liver disease as a comorbidity, and both of them suffered from bacterial infection. Other comorbidities such as diabetes, hypertension, heart disease, stroke, chronic kidney disease did not differ between the two categories. COVID-19 patients with bacterial infections had a longer hospital length of stay, a higher proportion of respiratory failure, ICU treatment, ventilator use.

**Table 2.** Comparison of COVID-19 patients who had bacterial infections and without bacterial infections.

	Bacterial Infection		No Bacterial Infection		Total (n.%)		p value
	43		175		218	100	
**Sex**							
Male (n=35)	20	29.85	100	45.90	120.00	75.75	0.21
Female (n=32)	23	34.33	75	34.40	98.00	68.73	
**Age(Mean/SD)**	55.28	12.98	51.76	14.72	52.45	14.44	0.153
**Comorbidities**							
HT (n.%)	18	8.30	47	21.60	65.00	29.90	0.054
DM (n.%)	15	6.90	60	27.50	75.00	34.40	0.94
Geriatric (> 60 years) (n.%)	12	5.50	47	21.60	59.00	27.10	0.89
Heart disease (n.%)	4	1.80	10	4.60	14.00	6.40	0.39
CKD (n.%)	2	0.90	7	3.20	9.00	4.10	0.85
Stroke (n.%)	1	0.50	10	4.60	11.00	5.10	0.36
COPD (%)	1	0.50	0	0.00	1.00	0.50	0.043
Liver disease (n.%)	1	0.50	0	0.00	1.00	0.50	0.043
Smoker (n.%)	1	0.50	6	2.80	7.00	3.30	0.42
**ICU room (n.%)**	22	10.1	14	6.4	36.00	16.50	<0.05
**Respiratory Failure (n.%)**	14	6.4	29	13.3	43.00	19.70	0.018
**Ventilator (n.%)**	9	4.1	14	6.4	23.00	10.50	0.013
**Sepsis (n.%)**	7	3.2	10	4.6	17.00	7.80	0.021
**Antibiotic use**	36	16.5	128	58.7	164.00	75.20	
**Length of stay (mean/SD)**	17.6	6.62	13.31	7.12			<0.05
**Outcome (between group)**							
Discharge (n.%)	35	81.40	148	84.57	183.00	165.97	0.04
Death (n.%)	7	16.28	14	8	21.00	24.28	

Mortality occurs in 16.28% of COVID-19 patients with bacterial infections, higher than those without bacterial infection (8%). Overall, COVID-19 patients with bacterial infection had a worse prognosis than those without bacterial infection (p<0.04).

**Figure 1.**
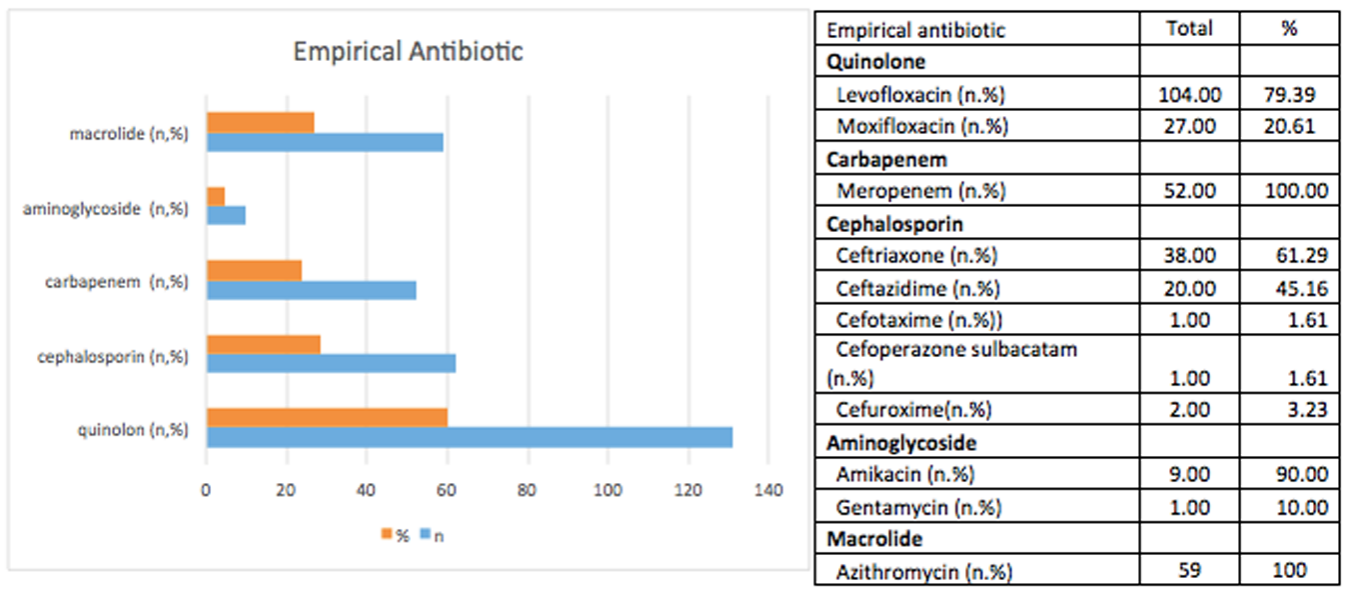
The empirical antibiotic use in study population.

The empirical antibiotic was given to 75.2% of the patients. Antibiotics used in these patients were quinolones (60.1%), cephalosporins (28.44%), carbapenem (23.85%), and aminoglycosides (4.59%). Quinolone used in these patients was mostly levofloxacin (79.39%), and the others were moxifloxacin (20.61%). The carbapenem used in this study was meropenem. Cephalosporins used were ceftriaxone, ceftazidime, cefotaxime, cefoperazone-sulbactam, and cefuroxime (see Figure 1).

### Pathogens associated with a bacterial infection

**Figure 2.**
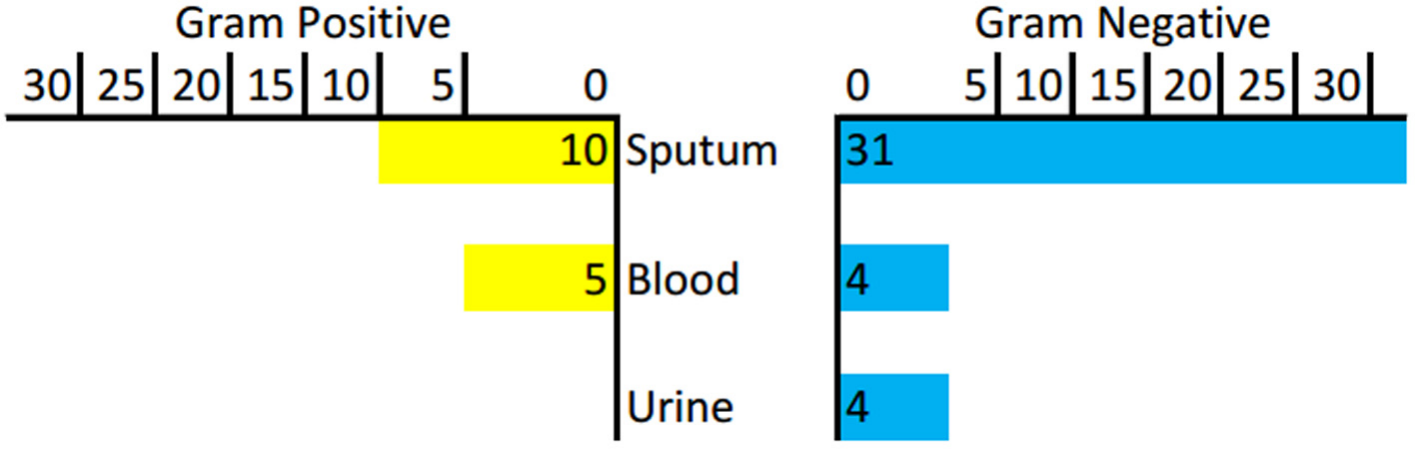
Distribution of Gram-positive and Gram-negative bacteria.

We collected 110 culture samples from suspected bacterial infection patients, consisting of 44 blood samples, nine urine samples, and 57 sputum samples. Among them, bacteria were detected in nine blood cultures, four urine cultures, and 47 sputum cultures. Sputum samples were collected, some from spontaneous sputum (n=47) and the others from endotracheal tube aspirate (n=10). Gram-negative bacteria dominate the culture result (70.37%). Bacteria found in the blood were extended-spectrum -lactamase (ESBL)-producing *Klebsiella pneumoniae* (2), *Pseudomonas fluorescens* (1), *Pseudomonas putida* (1), *Staphylococcus epidermidis* (2), *Staphylococcus haemolyticus* (1), *Staphylococcus hominis* (2). Bacteria found in urine were ESBL-producing *Escherichia coli* (2), *Enterococcus faecalis* (1), and *Pseudomonas putida* (1). There were 47 isolates found in sputum cultures, in which six of them were fungi ( *Candida spp*). The most frequent bacteria found was *Acinetobacter baumanni*i (9), followed by *Klebsiella pneumoniae* (5), *Pseudomonas aeruginosa* (4), *Escherichia Coli* (2), *Enterobacter cloacae complex* (3), and *Staphylococcus haemolyticus* (3) (see Table 3 and Figure 2).

**Table 3.** Result of microbiological culture.

Culture	Number
**Sputum**	57
Acinetobacter baumannii	9
Candida spp.	6
Klebsiella pneumoniae	5
Pseudomonas aeruginosa	4
Enterobacter cloacae complex	3
Staphylococcus haemolyticus	3
Streptococcus mitis / Streptococcus oralis *	3
Escherichia coli	2
Escherichia coli (ESBL)	1
Others	11
No growth	10
**Blood**	44
Klebsiella pneumoniae (ESBL+)	2
Staphylococcus epidermidis *	2
Staphylococcus hominis *	2
Pseudomonas flurescens	1
Pseudomonas putida	1
Staphylococcus haemolyticus	1
No growth	35
**Urine**	9
Escherichia coli (ESBL)	2
Enterococcus faecalis	1
Pseudomonas putida	1
No growth	5

Note : *, considered as normal flora bacteria.

## Discussion

Our study focuses on bacterial infection results in COVID-19 patients and evaluates their clinical and microbiological features. Clinical characteristics of the study group were described according to disease severity. According to disease severity, the number of patients with moderate, severe, and critically ill manifestations were 126 (57.8%), 40 (18.3%), and 52 (23.9%), respectively. The average age was 52.45 (14.44) years, and 55.05% of patients were male. Other studies regarding the characteristic of hospitalized COVID-19 patients showed various results. Zhou *et al.* reported that the median age of the 191 patients was 56.0 years (1887 years), and most patients were male ^12^. Lv *et al.* reported 354 hospitalized patients, 175 (49.44%) were male, and the median age was 62 years (2390 years) ^13^. A study conducted in Saudi Arabia reported among the 99 hospitalized patients, the median age was 44 years (range 1987), and the majority were men (66%) ^14^. We found that diabetes and hypertension were the most common comorbidity, similar to these other studies ^12,15^.

In this study, inflammation signs such as leucocyte count, neutrophil count, procalcitonin in the critically ill group were higher than the others. Lymphopenia in COVID-19 patients occurs through various mechanisms such as direct virus invasion lymphocytes, lymphatic organ destruction, altered inflammatory cytokines production leading to lymphocyte apoptosis, and inhibiting lymphocytes function by metabolic molecules produced by metabolic disorders, such as hyperlactic academia ^16^. Neutrophil and lymphocyte ratio (NLR) has been proposed as a prognostic marker of severity in various chronic inflammatory diseases, including cardiovascular diseases and oncological processes ^17^.

Our result showed a significant increase of procalcitonin level and a higher proportion of bacterial infection in severe and critically ill COVID-19 patients. This finding is consistent with the study result from Wang *et al.* in elderly COVID-19 patients. They also concluded that bacterial infection was also considered as a predictor of mortality in these patients ^18^. Increased procalcitonin level in COVID-19 patients may be associated with the release of some cytokines, especially IL-6. It is established that procalcitonin is a better marker to predict severity, prognosis, or the sepsis course and is also helpful to guide antibiotic usage. Increased procalcitonin in critically ill patients with COVID-19 can represent a bacterial co-infection, and blood cultures for bacteria detection are needed to a prompt response ^19^.

In this study, we found that bacterial infection was confirmed in 43 patients (19,7%). The bacterial infection that was detected at patient admission (bacterial co-infection) was 23%, while bacterial infection that occurs late during hospital stay (secondary infection) was 77%. The prevalence of bacterial co-infections in patients admitted to the ICU for acute respiratory failure related to COVID-19 pneumonia is poorly studied ^20, 21^. In Fu *et al*. study, 13.9% (5 of 36) of the patients in the ICU were diagnosed with COVID-19 and secondary bacterial infection. In another report that was published from a UK secondary care setting, 27 among 836 patients (3.2%) had early confirmed bacterial isolates identified (05 days post-admission), rising to 51 cases (6.1%) during the admission. In a study conducted in Shiraz, Iran, in 2009, Hassanzadeh *et al*. suggested that ICU-acquired infections were documented in 51.7% of ICU patients, with a mortality rate of 10.9% (5 patients) ^22, 23^. Our finding was relatively higher among other studies. The possible explanation is that most of the patients had been treated in hospital for more than two weeks (average 1217 days). Secondary infections usually correspond with nosocomial or healthcare-associated infections. Nosocomial infections are most commonly correlated with invasive medical devices or surgical procedures. Lower respiratory tract and bloodstream infections have the highest mortality, while urinary tract infections are the most common ^24^.

The median length of stay among patients in our study was higher in the bacterial co-infection group rather than non co-infection, with an average of 17.6 and 13.31 days, respectively. This finding is consistent with the result of several studies ^22, 25^. Sharifpour *et al.* reported that the median length of stay is around 15 days (interquartile range, 2 to 39). A study on respiratory co-infection in patients with pandemic 2009 influenza A (H1N1) virus infection showed that ICU length of stay was three days longer among patients with co-infection. Another study by Zhou *et al.* reported a longer length of stay of 8.0 days (4.012.0) of all patients with COVID-19 admitted to their ICU ^12^. These findings suggest that the length of ICU stay can be prolonged if patients become co-infected.

The most common bacterial infection in this study was from respiratory tract infection, followed by bloodstream infection and urinary tract infection. The culture result was dominated by gram-negative bacteria (75.68%). This finding is similar to the result from Zhang *et al.* (50%), although they also included virus and fungal cultures ^3^. Gram-negative bacteria were reported responsible for more than 30% of healthcare-associated infections, and these bacteria predominate in cases of ventilator-associated pneumonia (47%) and urinary tract infections (45%) ^24^. In intensive care units (ICUs), gram-negative bacteria account for about 70% of these types of infections ^26^. Gram-negative bacteria are highly efficient at up-regulating or acquiring genes that code for antimicrobial resistance mechanisms, especially in the presence of antibiotic selection pressure. Among gram-negative bacteria, the *Enterobacteriaceae* family being the most commonly found and multidrug-resistant organisms, including *Pseudomonas aeruginosa*, *Acinetobacter baumannii*, and extended-spectrum -lactamase (ESBL)producing or carbapenemase *-*producing *Enterobacteriaceae*, are increasingly being reported worldwide ^24^. The identification of bacterial infection with gram-negative organisms is more reflected as a complication of ICU care and is not suggested as a specific predilection for co-infections in COVID-19 ^23^.

*Acinetobacter baumannii* is the most common bacteria found in this study. Several reports regarding bacterial infection in COVID-19 also found a similar result ^3, 7, 9, 19, 24^. *Acinetobacter baumannii* is the common cause of respiratory tract infection, especially in ICU settings where patients often received mechanical ventilation. Environmental contamination also plays a role in this phenomenon. Our ICUs for COVID-19 patients were set as a large ward consist of 16 beds. Although we already managed the distance between beds for more than 1.5 meters, the inter-patient transmission is hard to avoid. It is crucial to maintain hand hygiene compliance of the staff, strict cleanliness, and disinfection of the hospital environment in the hospital, especially in high-care or ICU setting.

Antibiotic is often given to COVID-19 patients for various reasons. The clinical manifestation on presentation was sometimes challenging to distinguish with bacterial infection ^2^. Secondary infection and nosocomial infection are also other considerations in the use of antibiotics. In this study, 75.3% of patients included were given antibiotics. A meta-analysis conducted about bacterial co-infection and secondary infection in COVID-19 patients reported that the majority of patients with COVID-19 received antibiotics (71.9%, 95%CI 56.1 to 87.7%) ^27^. Another meta-analysis reported that >90% of COVID-19 patients were given an empirical antibiotic, while the bacterial infection was detected only in 7% of hospitalized patients and 14% of ICU patients ^28^. Quinolones (levofloxacin and moxifloxacin) were frequently prescribed, followed by cephalosporins and carbapenems. Indonesian national guidelines for COVID-19 treatment recommend using antibiotics in severe and critically ill patients, especially if there was suspicion of bacterial infection. The empirical antibiotic choice was intravenous azithromycin or levofloxacin ^10^. In our hospital, levofloxacin is more feasible than azithromycin, so clinicians prefer to prescribe this drug. In our hospital, antibiotic use usually was decided based on clinical signs and laboratory parameters (leukocytosis, increased CRP, or procalcitonin). Culture examinations were not routinely performed, preferably in ICU/high-care settings and in patients showing bacterial infection.

The widespread use of antibiotics in this pandemic era raises concern about antimicrobial resistance ^4, 6^. Although bacterial infection in COVID-19 patients has already been reported in various results, evidence supports the restrictive use of antibiotics. Clinical guidelines and standard testing to diagnose bacterial infection in COVID-19 are not clearly available. The microbiological examination is an important strategy to confirm bacterial infection and decide the antibiotic choice ^4^. Sputum, blood culture samples, and also pneumococcal urinary antigen testing should also be performed. Antibiotics should be stopped in patients who represent cultures, and urinary antigen tests show no signs of bacterial pathogens after 48 hours ^29^. 

There are some limitations to our study. First, we only included COVID-19 patients in high-care and intensive care units. In low-care settings, bacterial infection cannot be confirmed due to limited data. Second, we only reported bacterial infection from microbiology results. Virus culture or gene sequencing to detect pathogens is not available in our hospital. Bacterial culture was not performed in all COVID-19 patients, so in a condition where the sign of infection is absent such as in elderly or in immunocompromised patients, the bacterial infection might be underdiagnosed.

## Conclusions

Bacterial infection was detected in 19.7% of COVID-19 patients admitted in high-care and intensive care units, predominantly secondary infections. COVID-19 patients who suffered from bacterial infection have a longer length of stay and have higher mortality. The pathogen commonly found in this study was *Acinetobacter baumannii* that yielded from sputum. Increased antibiotic use and multi-drug resistant organism is an emerging problem in this pandemic situation. Infection control practice need to strictly conducted to reduced secondary or healthcare-associated infection

## Data availability

### Underlying data

Dryad: The clinical impact of bacterial co-infection among moderate, severe and critically ill COVID-19 patients in the second referral hospital in Surabaya, https://doi.org/10.5061/dryad.sxksn0328 ^30^.

This project contains the following underlying data:

- Baseline characteristics for all patients
- Lab data for all participants

Data are available under the terms of the Creative Commons Zero No rights reserved data waiver (CC0 1.0 Public domain dedication).
