# Supplementary material for: The clinical impact of bacterial co-infection among moderate, severe and critically ill COVID-19 patients in the second referral hospital in Surabaya
Source: F1000Res. 2021 Mar 29;10:113. Originally published 2021 Feb 15. [Version 2] doi: 10.12688/f1000research.31645.2 (PMC8030114; doi:10.12688/f1000research.31645.2)
Supplement: Peer review history

# Peer review: Reviewer response for version 2

**DOI:** 10.5256/f1000research.55357.r82430

**Published:** 2021-04-07

**Authors:** Fitriana Murriya Ekawati

**Affiliations:** 1 Department of Family and Community Medicine, Universitas Gadjah Mada, Yogyakarta, Indonesia

I can see that there are some improvements in the manuscript that the authors have provided. However, I believe the quality can be further improved by accomodating these minor suggestions:

1. Abstract, background, remove **"in our country Indonesia"the sentence can be improved as: While the prevalence of COVID in Indonesia is high, data on the prevalence of...are limited.
2. The information for the antibiotic selection is already in the heading of "pathogens associated with bacterial infection", I wonder if the last paragraph on the "bacterial infection" can be shifted under the "pathogens"heading?
3. Discussion: what are suggestions for further research raised from this paper? There are some uncertainties mentioned by the authors regarding the use/guideline of antibiotics in COVID. It can be one of them.

Is the work clearly and accurately presented and does it cite the current literature?

Yes

If applicable, is the statistical analysis and its interpretation appropriate?

Yes

Are all the source data underlying the results available to ensure full reproducibility?

Yes

Is the study design appropriate and is the work technically sound?

Yes

Are the conclusions drawn adequately supported by the results?

Yes

Are sufficient details of methods and analysis provided to allow replication by others?

Yes

Reviewer Expertise:

Health service research in LMICs

I confirm that I have read this submission and believe that I have an appropriate level of expertise to confirm that it is of an acceptable scientific standard.

# Peer review: Reviewer response for version 1

**DOI:** 10.5256/f1000research.34701.r81317

**Published:** 2021-03-23

**Authors:** Erni J. Nelwan

**Affiliations:** 1 Division of Tropical and Infectious Disease, Universitas Indonesia, Jakarta, Indonesia

A well written article and proper data collection to support the conclusion. From a LMIC like Indonesia, this article shares important findings, since the co-bacterial infection is not more than 20%, higher than reported in IDSA data but lower compared to what had been expected in clinical practice among Indonesian doctors.

 Authors might add value by putting a highlight on what is the clinical appearance of the co-bacterial infected vs. the non co-bacterial infected for readers to apply in daily practice.

Is the work clearly and accurately presented and does it cite the current literature?

Yes

If applicable, is the statistical analysis and its interpretation appropriate?

Yes

Are all the source data underlying the results available to ensure full reproducibility?

Yes

Is the study design appropriate and is the work technically sound?

Yes

Are the conclusions drawn adequately supported by the results?

Yes

Are sufficient details of methods and analysis provided to allow replication by others?

Yes

Reviewer Expertise:

tropical medicine and infectious disease

I confirm that I have read this submission and believe that I have an appropriate level of expertise to confirm that it is of an acceptable scientific standard.

# Peer review: Reviewer response for version 1

**DOI:** 10.5256/f1000research.34701.r80840

**Published:** 2021-03-09

**Authors:** Fitriana Murriya Ekawati

**Affiliations:** 1 Department of Family and Community Medicine, Universitas Gadjah Mada, Yogyakarta, Indonesia

Thank you very much for the opportunity for reviewing this manuscript. This manuscript has presented interesting findings of bacterial coinfection in COVID-19 patients. Data collected for the research were from patients' medical records in Univ Airlangga hospital in Indonesia.

 The presentations of Methods, Results, and Discussion in the manuscript are appropriate. However, there are some minor revisions needed, which I believe are beneficial for improving the manuscript:

- Introduction

- Line 1. I would recommend the authors to improve the first sentence of the first and second paragraph to improve clarity.

 - Are there any prior investigations related to this topic in Indonesia? Please write/present if in the introduction there is any.

- Methods

- Just to clarify, so the infection was examined if there is any suspicion of bacterial infection and not a routine examination?

 - Statistical analysis: SPSS version 24.0 (citation?).

- Results

- Patient characteristic and laboratory findings, is there any table/summary table associated with the patients' leucocyte count, procalcitonin? Please cite/involve the summary table in the main manuscript.

- Discussion

- Are there any things that still unknown/need further investigation arising from this research?

 - What are the study implications for further research and practice?

 - I would recommend a language proofreading for this manuscript.

 Best wishes to the authors for the manuscript revision.

Is the work clearly and accurately presented and does it cite the current literature?

Yes

If applicable, is the statistical analysis and its interpretation appropriate?

Yes

Are all the source data underlying the results available to ensure full reproducibility?

Yes

Is the study design appropriate and is the work technically sound?

Yes

Are the conclusions drawn adequately supported by the results?

Yes

Are sufficient details of methods and analysis provided to allow replication by others?

Yes

Reviewer Expertise:

Health service research in LMICs

I confirm that I have read this submission and believe that I have an appropriate level of expertise to confirm that it is of an acceptable scientific standard, however I have significant reservations, as outlined above.

# Response

**Published:** 2021-03-09

**Authors:** Tri Pudy Asmarawati

**Affiliations:** Department of Internal Medicine, Faculty of Medicine, Universitas Airlangga, Hospital, Indonesia

We firstly would like to thank you for the kindly comprehensive review of our manuscript. Here's to answer and respond to your questions:

 1. Introduction

- We believe that there is no prior investigation linked to this topic ever done before in Indonesia

2. Methods

- We clarify that the bacterial culture examination was done on the suspicious patients only.

3. Results

- We have enclosed the leucocyte and procalcitonin data of the patients on the table 1.

4. Discussion

- In the study to come, further investigation can probably be done on the low health-care setting, also bigger concerns on under-diagnosed bacterial co-infection of elderly and immunocompromised patients can be taken into consideration.
- The implication of the study is that we find the bacterial culture examination necessary in the COVID-19 patients management since the bacterial co-infection might worsen the clinical outcome.

Furthermore, we also appreciate your suggestions in order to improve the final quality of this manuscript. We are currently working on the minor revisions, proof-reading, and going to submit the latest version promptly.

# Response

**Published:** 2021-03-25

**Authors:** Tri Pudy Asmarawati

**Affiliations:** Department of Internal Medicine, Faculty of Medicine, Universitas Airlangga, Hospital, Indonesia

We have just process revised several points on the manuscript as what the reviewer, named Fitriana Murriya Ekawati, had recommended a couple days ago. We include the latest version of our manuscript down below along with highlighted changes. We also insert our reply to the reviewer, which said:

 We firstly would like to thank you for the kindly comprehensive review of our manuscript. Here's to answer and respond to your questions:

 1. Introduction

 We believe that there is no prior investigation linked to this topic ever done before in Indonesia. We added this information in the second and last paragraphs of Introduction

 2. Methods

 Yes, the bacterial culture examination was done on the suspicious patients only. Please refer to the last sentence of the paragraph 8 in the Discussion section.

 3. Results

 We have enclosed the leucocyte and procalcitonin data of the patients on the table 1 on Result section

 4. Discussion

 In the study to come, further investigation can probably be done in the low health-care setting, also bigger concerns on under-diagnosed bacterial co-infection of elderly and immunocompromised patients can be taken into consideration. we added this sentence on the last paragraph of the discussion.

 The implication of the study is that we find the bacterial culture examination necessary in the COVID-19 patients management since the bacterial co-infection might worsen the clinical outcome. Therefore this examination should be conducted when the patient shows signs of infection during the intensive care. We added this sentence in the second paragraph of the introduction.

 Furthermore, we also appreciate your suggestions in order to improve the final quality of this manuscript. We are currently working on the minor revisions, proof-reading, and going to submit the latest version it promptly

 Hope this will meet all the requirement and we will receive the final pdf proof soon.

 Thanks in advance,

 Tri Pudy Asmarawati and research team
